# Prognostic Value of Histone Acetyl Transferase 1 (HAT-1) and Inflammatory Signatures in Pancreatic Cancer

**DOI:** 10.3390/cimb46050239

**Published:** 2024-04-25

**Authors:** Miguel A. Ortega, Laura Jiménez-Álvarez, Oscar Fraile-Martinez, Cielo Garcia-Montero, Luis G. Guijarro, Leonel Pekarek, Silvestra Barrena-Blázquez, Ángel Asúnsolo, Laura López-González, María Del Val Toledo-Lobo, Melchor Álvarez-Mon, Miguel A. Saez, Alberto Gutiérrez-Calvo, Raúl Díaz-Pedrero

**Affiliations:** 1 Department of Medicine and Medical Specialities (CIBEREHD), Faculty of Medicine and Health Sciences, University of Alcala, 28801 Alcala de Henares, Madrid, Spain; laura.jimenezal@gmail.com (L.J.-Á.); oscar.fraile@uah.es (O.F.-M.); cielo.garcia@uah.es (C.G.-M.); silvebarrena@gmail.com (S.B.-B.); mademons@gmail.com (M.Á.-M.); msaega1@oc.mde.es (M.A.S.); 2 Ramón y Cajal Institute of Sanitary Research (IRYCIS), 28034 Madrid, Spain; luis.gonzalez@uah.es (L.G.G.); leonel.pekarek@gmail.com (L.P.); angel.asunsolo@uah.es (Á.A.); laura.lgonzalez@uah.es (L.L.-G.); mval.toledo@uah.es (M.D.V.T.-L.); raul.diazp@uah.es (R.D.-P.); 3 Cancer Registry and Pathology Department, Principe de Asturias University Hospital, 28806 Alcala de Henares, Madrid, Spain; 4 Department of General and Digestive Surgery, General and Digestive Surgery, Principe de Asturias University Hospital, 28806 Alcala de Henares, Madrid, Spain; agutierrezcalvo@telefonica.net; 5 Unit of Biochemistry and Molecular Biology, Department of System Biology (CIBEREHD), University of Alcala, 28801 Alcala de Henares, Madrid, Spain; 6 Oncology Service, Guadalajara University Hospital, 19002 Guadalajara, Spain; 7 Department of Surgery, Medical and Social Sciences, Faculty of Medicine and Health Sciences, University of Alcala, 28801 Alcala de Henares, Madrid, Spain; 8 Department of Epidemiology and Biostatistics, Graduate School of Public Health and Health Policy, University of New York, New York, NY 10012, USA; 9 Unit of Cell Biology, Department of Biomedicine and Biotechnology, University of Alcala, 28801 Alcala de Henares, Madrid, Spain; 10 Immune System Diseases-Rheumatology, Oncology Service and Internal Medicine (CIBEREHD), Principe de Asturias University Hospital, 28806 Alcala de Henares, Madrid, Spain; 11 Pathological Anatomy Service, Central University Hospital of Defence-UAH Madrid, 28801 Alcala de Henares, Madrid, Spain

**Keywords:** pancreatic ductal adenocarcinoma (PDAC), prognostic biomarkers, histone acetyl transferase 1 (HAT-1), interleukin 10 (IL-10)

## Abstract

Pancreatic cancer is a type of gastrointestinal tumor with a growing incidence and mortality worldwide. Pancreatic ductal adenocarcinoma (PDAC) constitutes 90% of cases, and late-stage diagnosis is common, leading to a 5-year survival rate of less than 10% in high-income countries. The use of biomarkers has different proven translational applications, facilitating early diagnosis, accurate prognosis and identification of potential therapeutic targets. Several studies have shown a correlation between the tissue expression levels of various molecules, measured through immunohistochemistry (IHC), and survival rates in PDAC. Following the hallmarks of cancer, epigenetic and metabolic reprogramming, together with immune evasion and tumor-promoted inflammation, plays a critical role in cancer initiation and development. In this study, we aim to explore via IHC and Kaplan–Meier analyses the prognostic value of various epigenetic-related markers (histones 3 and 4 (H3/H4), histone acetyl transferase 1 (HAT-1), Anti-Silencing Function 1 protein (ASF1), Nuclear Autoantigenic Sperm Protein (NASP), Retinol Binding Protein 7 (RBBP7), importin 4 (IPO4) and IPO5), metabolic regulators (Phosphoglycerate mutase (PGAM)) and inflammatory mediators (allograft inflammatory factor 1 (AIF-1), interleukin 10 (IL-10), IL-12A and IL-18) in patients with PDAC. Also, through a correlation analysis, we have explored the possible interconnections in the expression levels of these molecules. Our results show that higher expression levels of these molecules are directly associated with poorer survival rates in PDAC patients, except in the case of IL-10, which shows an inverse association with mortality. HAT1 was the molecule more clearly associated with mortality, with a hazard risk of 21.74. The correlogram demonstrates an important correlation between almost all molecules studied (except in the case of IL-18), highlighting potential interactions between these molecules. Overall, our study demonstrates the relevance of including different markers from IHC techniques in order to identify unexplored molecules to develop more accurate prognosis methods and possible targeted therapies. Additionally, our correlation analysis reveals potential interactions among these markers, offering insights into PDAC’s pathogenesis and paving the way for targeted therapies tailored to individual patient profiles. Future studies should be conducted to confirm the prognostic value of these components in PDAC in a broader sample size, as well as to evaluate the possible biological networks connecting them.

## 1. Introduction

Pancreatic cancer is a type of gastrointestinal tumor with a growing incidence and mortality worldwide. According to epidemiological data, the incidence of pancreatic cancer increased from 5.0 per–100,000 person-years in 1990 to 5.7 per–100,000 person-years in 2017 [1]. However, recent epidemiological data show an annual increase of 1.1% globally, with a global incidence of 18.6 per–100,000 person-years projected by 2050 [2]. Currently, pancreatic cancer ranks as the fourth leading cause of cancer deaths in Europe and the United States [3], but it is expected to become the second leading cause of cancer-related mortality in the future [4,5]. Pancreatic ductal adenocarcinomas (PDACs) account for up to 90% of all cases of pancreatic tumors [6]. Most cases of pancreatic tumors are diagnosed at late stages due to difficulties in early detection, the biological properties of this type of cancer and a very poor 5-year survival rate of below 10% in high-income countries [2].

The study and exploration of serological and tissue biomarkers are a pivotal part of pancreatic cancer research, offering different translational applications such as early diagnosis, accurate prognosis or potential therapeutic targets to be explored [7,8]. In this sense, a number of studies have consistently demonstrated a correlation between the level of tissue expression of different molecules measured via immunohistochemistry (IHC) and survival rates in PDAC patients [9,10,11,12,13,14], aiding to predict the progression of the disease and explore possible therapeutic alternatives. Therefore, the search and establishment of different relevant tissue markers highlight the connection between biological and clinical variables in different types of tumors such as PDAC, elucidating the critical importance of these types of studies.

Hanahan and Weinberg [15] described the hallmarks of cancer, reporting the central features of tumoral cells. The relevance of the immune system and inflammation along with the metabolic reprogramming is clearly established in their works. Recently, in the latest update of the hallmarks of cancer, Hanahan added non-mutational epigenetic reprogramming as a novel but also a critical point of study in tumoral cells [16]. Because of this, inflammatory and epigenetic biomarkers have demonstrated their prognostic relevance in different types of cancer, including PDAC [17,18,19,20]. In this context, our study aims to further explore the possible prognostic value of various epigenetic-related markers (histones 3 and 4 (H3/H4), histone acetyl transferase 1 (HAT-1), Anti-Silencing Function 1 protein (ASF1), Nuclear Autoantigenic Sperm Protein (NASP), Retinol Binding Protein 7 (RBBP7), importin 4 (IPO4) and IPO5), metabolic regulators (Phosphoglycerate mutase (PGAM)) and inflammatory mediators (allograft inflammatory factor 1 (AIF-1), interleukin 10 (IL-10), IL-12A and IL-18) in patients with PDAC. With this purpose, we analyzed the protein expression of these components in 41 patients diagnosed with PDAC using IHC techniques. Subsequently, through Kaplan–Meier curves and additional statistical methods, we aimed to delineate the potential correlations between the expression levels of these proteins and patient survival, investigating whether these biological variables are interconnected in any manner and thereby establishing a molecular network that connects our findings.

## 2. Materials and Methods

### 2.1. Study Design and Sample Collection

Paraffin-embedded pancreatic tissue sections from 41 patients with ductal adenocarcinoma who underwent pancreatoduodenectomy surgery (curative resection) were used in our study. The patients were monitored for 60 months. The diagnosis adhered to a past work’s guidelines [21]. The current investigation was planned as a retrospective, analytical, observational cohort study with long-term follow-up. Retrospective reviews were conducted of the paraffin blocks, various details containing comprehensive clinical information on the patients, and the follow-up data.

This study was developed in compliance with Good Clinical Practice guidelines, the principles outlined in the most recent Declaration of Helsinki (2013), and the Convention of Oviedo (1997). It was conducted in accordance with the fundamental ethical principles of autonomy, beneficence, non-maleficence, and distributive justice. The information and data gathered conformed with the most recent data protection laws, which include Regulation (EU) 2016/679 and Organic Law 3/2018 of December 5, which protects personal data and guarantees digital rights.

### 2.2. Immunohistochemical Analysis

Pancreatic tissue samples fixed in paraffin were subjected to immunohistochemical investigations. The protocol requirements (Table 1) included a description of the antibody recovery stage. Following known methods, antigen/antibody responses were identified using the avidin–biotin (ABC) complex technique in conjunction with avidin–peroxidase [22]. Samples were incubated with a 3% BSA blocker (Catalog #37525; Thermo Fisher Scientific, Inc., Waltham, MA, USA) and PBS overnight at 4 °C following an hour and thirty minutes of incubation with the primary antibody. After being incubated for 90 min at room temperature with a biotin-conjugated secondary antibody, the samples were diluted in PBS (rabbit IgG, diluted 1/300 (RG-96; Sigma-Aldrich, St. Louis, MO, USA), goat IgG, diluted 1/100 (GT-34/B3148; Sigma-Aldrich), and mouse IgG, diluted 1/300 (F2012/045K6072; Sigma-Aldrich)). ExtrAvidin^®^-Peroxidase (Sigma-Aldrich; Merck KGaA, Darmstadt, Germany), a conjugate of avidin and peroxidase, was utilized for 60 min at RT (1:200 with PBS). A Chromogenic Diaminobenzidine (DAB) Substrate Kit (cat. no. SK-4100; Maravai LifeSciences, San Diego, CA, USA) was then used to measure protein expressions. It was set up just before exposure and included five milliliters of distilled water, two drops of buffer, four drops of DAB, and two drops of hydrogen peroxide. Brown staining could be observed when the chromogenic peroxidase substrate produced a signal for 15 min at room temperature. To detect each protein, sections of the same tissue were designated as negative controls. PBS was used as a blocking solution instead of being incubated with the primary antibody. Carazzi hematoxylin was used to contrast each tissue for 15 min at room temperature.

### 2.3. Histopathological Evaluation

A Zeiss Axiophot light microscope (Carl Zeiss, Oberkochen, Germany) with an AxioCam HRc digital camera (Carl Zeiss, Oberkochen, Germany) was used to view tissue slices. Owing to the significance of the proteins under investigation, the histological assessment was conducted based on the immunohistochemistry staining intensity using Score. Thus, using the IRS Score approach, histological samples from individuals diagnosed with pancreatic cancer were categorized as low/medium (1/2), high (3), and negative expression (0) [9,23]. In each of the five parts, seven randomly chosen microscopy fields were evaluated for every set of patients.

When the mean proportion of the labeled sample was greater than or equivalent to 5% of the entire sample, the individuals were categorized as positive. As previously described in previous works [24], this was accomplished by computing the overall percentage of marked tissue in each microscope field to produce an average of the research sample. Two researchers worked independently to observe and quantify the samples.

### 2.4. Analytical Statistics

First, a normality test (Kolmogorov–Smirnov, all *p* < 0.001) was performed for all the markers. We found that they did not follow a normal distribution; hence, non-parametric tests were required to provide medians and interquartile ranges to explain the data (a Mann–Whitney U test). In order to assess the correlation between clinicopathological and immunohistochemical characteristics, survival comparisons using Kaplan–Meier curves and a logarithmic rank test were performed. A univariate analysis and a Cox proportional hazards regression analysis were performed to investigate the relationship between the observed prognosis of the variables and the immunohistochemistry parameters under investigation. The statistical software SPSS 22.0 (SPSS Inc., Chicago, IL, USA) was used for all analyses. For correlation analysis, statistical software R 4.3.3 was used, applying the package corrplot. Matrix of correlation was given with Spearman correlation coefficients, whose significancy was added with the corresponding cor.test. We used the package Hmisc to obtain the Spearman correlation matrix, and we used ggpairs to plot the pairwise scatterplots. We used the packages ggplot and reshape2 to finally express the results of correlation as correlation coefficients with numbers and color gradients. We assigned *p*-values to each pair of variables and applied false discovery rate corrections (fdr) with the function *p*.adjust.

## 3. Results

### 3.1. Clinical and Sociodemographic Features of the Patients

A total of 41 patients (27 men and 14 women, median age = 72.00 [45.00–88.00] years) were evaluated in an observational, longitudinal, analytical, and retrospective cohort study. The clinical and sociodemographic characteristics are collected in Table 2. As shown, 65.85% of the patients were men, 43.9% smoke, 26.83% had established drinking habits, 4.88% of the patients are obese, 55.56% had type 2 diabetes, 9.76% had chronic illnesses, and 26.83% have had malignant neoplasms in the past.

On the other hand, 13 patients had stage IV tumors, whereas the remaining 28 patients had stage < IV tumors (Table A1). The patients’ median expressions for CEA, AFP, and Ca19.9 were 2.32 [1.46–4.39] ng/mL, 5.43 [2.71–11.31] ng/mL, and 102.10 [44.91–805.00] U/mL, respectively (Table A1). The mean survival time for individuals with pancreatic cancer was 8.00 [2.98–13.02] months.

### 3.2. Patients with Pancreatic Cancer and High Expression Levels of Epigenetic-Related Markers Have Reduced Survival

Firstly, we explored the tissue expression of different epigenetic markers (HAT-1, IPO4, IPO5 NASP, RBBP7, H3, H4, ASF-1), classifying patients into high, low/median and negative expression for each protein. Then, through Kaplan–Meier curves, we analyzed the median survival for each group and the hazard ratio associated with the high-expression group. In Table A2, we summarize all these values for each protein.

**Figure 1:**
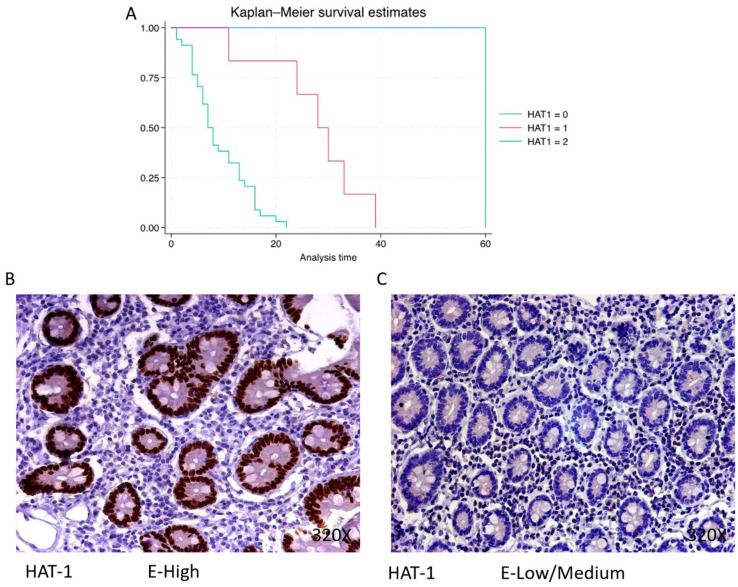
(**A**) Kaplan–Meier curves for survival time according to protein expression of HAT-1. Blue curve = negative expression; red curve = low/medium expression; green curve = high expression. (**B**,**C**) Images showing histopathological expression of HAT-1 in patients diagnosed with pancreatic cancer. High expression of the protein corresponds to an IRS score of 3, whereas low/medium expression corresponds to an IRS score of 1/2.

HAT-1 exhibits a predominantly high expression in 82.93% of PDAC cases, associated with a median survival of 7.5 (5–13) months. The hazard ratio of 21.743, along with its 95% confidence interval [2.909, 162.544], signifies a significant impact on survival (*p*-value = 0.003 **). Low/moderate expression is observed in 14.63% of cases with a median survival of 29 (25–32.2) months, while negative expression is only observed in one patient (2.44% of the total) with a median survival of 60 months. Kaplan–Meier survival estimates of HAT-1 and examples of high versus low/medium expression are summarized in Figure 1A–C.

**Figure 2:**
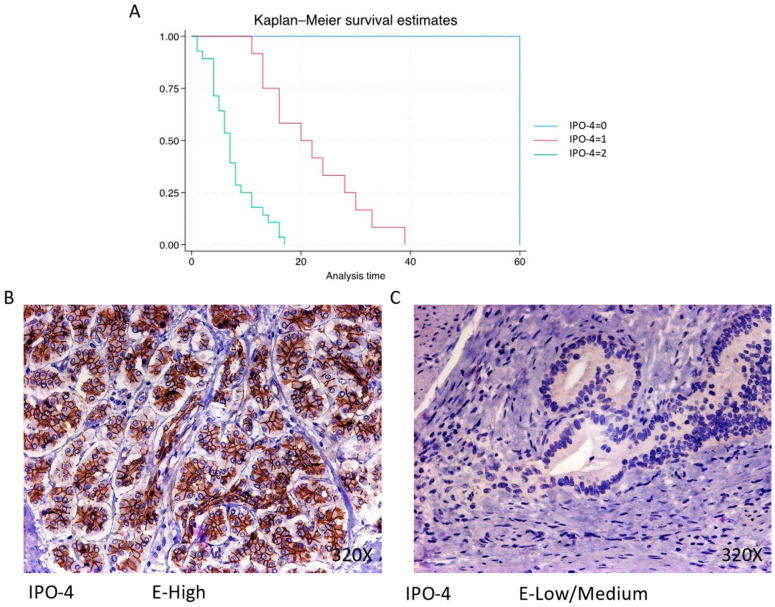
(**A**) Kaplan–Meier curves for survival time according to protein expression of IPO-4. Blue curve = negative expression; red curve = low/medium expression; green curve = high expression. (**B**,**C**) Images showing histopathological expression of IPO-4 in patients diagnosed with pancreatic cancer. High expression of the protein corresponds to an IRS score of 3, whereas low/medium expression corresponds to an IRS score of 1/2.

IPO-4 is expressed predominantly at a high level in 68.29% of cases, correlating with a median survival of 7 (4–9.5) months The hazard ratio of 8.094, with a 95% confidence interval [3.080, 21.267], indicates a substantial impact on survival (*p*-value < 0.001 ***). Negative expression is rare (2.44%), with a median survival of 60 months, while low/moderate expression is observed in 29.27% of cases, with a median survival of 21 months. Kaplan–Meier survival estimates of IPO-4 and examples of high versus low/medium expression are summarized in Figure 2A–C.

**Figure 3:**
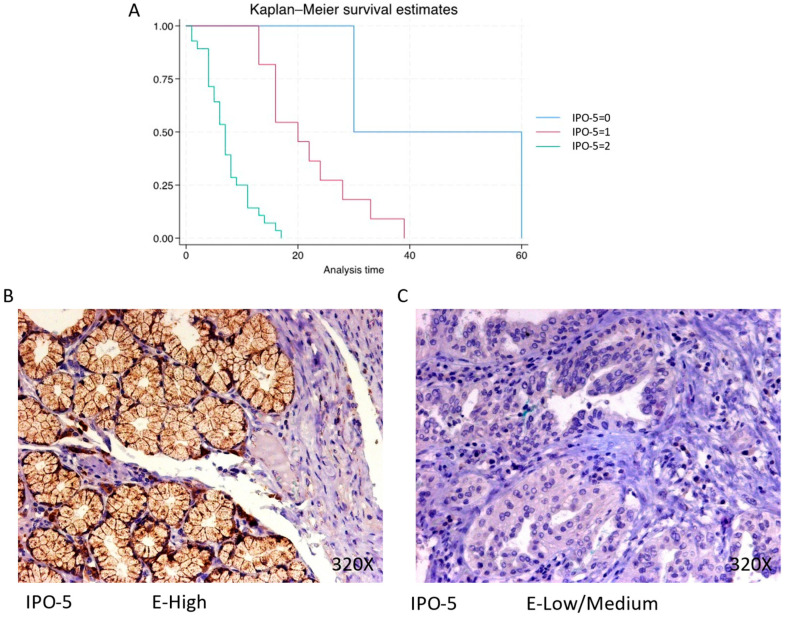
(**A**) Kaplan–Meier curves for survival time according to protein expression of IPO-5. Blue curve = negative expression; red curve = low/medium expression; green curve = high expression. (**B**,**C**) Images showing histopathological expression of IPO-5 in patients diagnosed with pancreatic cancer. High expression of the protein corresponds to an IRS score of 3, whereas low/medium expression corresponds to an IRS score of 1/2.

The expression of IPO-5 is predominantly high in 68.29% of PDAC cases, with a median survival of 7 (4–9.5) months. The hazard ratio of 7.493, along with a 95% confidence interval [3.067, 18.308], represents a significant impact on survival (*p*-value < 0.001 ***). Negative expression is observed in 4.88% of cases with a median survival of 45 months, while low/moderate expression is seen in 26.83% of cases with a median survival of 20 months. Kaplan–Meier survival estimates of IPO-5 and examples of high versus low/medium expression are summarized in Figure 3A–C.

**Figure 4:**
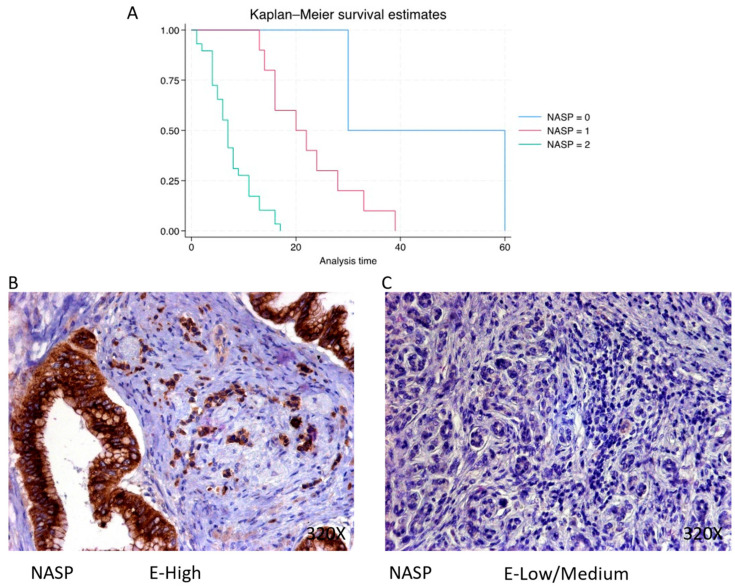
(**A**) Kaplan–Meier curves for survival time according to protein expression of NASP. Blue curve = negative expression; red curve = low/medium expression; green curve = high expression. (**B**,**C**) Images showing histopathological expression of NASP in patients diagnosed with pancreatic cancer. High expression of the protein corresponds to an IRS score of 3, whereas low/medium expression corresponds to an IRS score of 1/2.

NASP is predominantly expressed at a high level in 70.73% of cases, correlating with a median survival of 7 (4–11) months. The hazard ratio of 7.563, with a 95% confidence interval [2.927, 19.538], indicates a substantial impact on survival (*p*-value < 0.001 ***). Negative expression is observed in 4.88% of cases with a median survival of 45 months, while low/moderate expression is seen in 24.39% of cases with a median survival of 21 months. Kaplan–Meier survival estimates of NASP and examples of high versus low/medium expression are summarized in Figure 4A–C.

**Figure 5:**
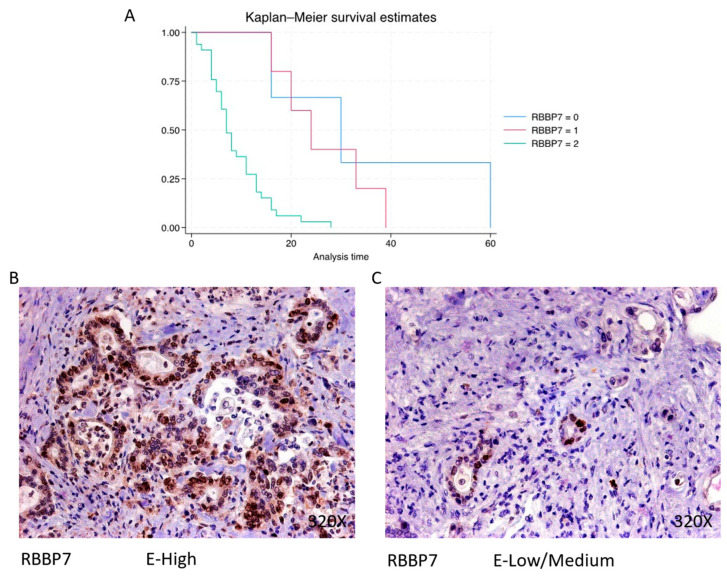
(**A**) Kaplan–Meier curves for survival time according to protein expression of RBBP7. Blue curve = negative expression; red curve = low/medium expression; green curve = high expression. (**B**,**C**) Images showing histopathological expression of RBBP7 in patients diagnosed with pancreatic cancer. High expression of the protein corresponds to an IRS score of 3, whereas low/medium expression corresponds to an IRS score of 1/2.

RBBP7 exhibits predominantly high expression in 80.49% of PDAC cases, associated with a median survival of 7 (5–13) months. The hazard ratio of 4.024, with a 95% confidence interval [1.788, 9.059], signifies a significant impact on survival (*p*-value < 0.001 ***). Negative expression is observed in 7.32% of cases with a median survival of 30 months, while low/moderate expression is seen in 12.2% of cases with a median survival of 24 months. Kaplan–Meier survival estimates of RBBP7 and examples of high versus low/medium expression are summarized in Figure 5A–C.

**Figure 6:**
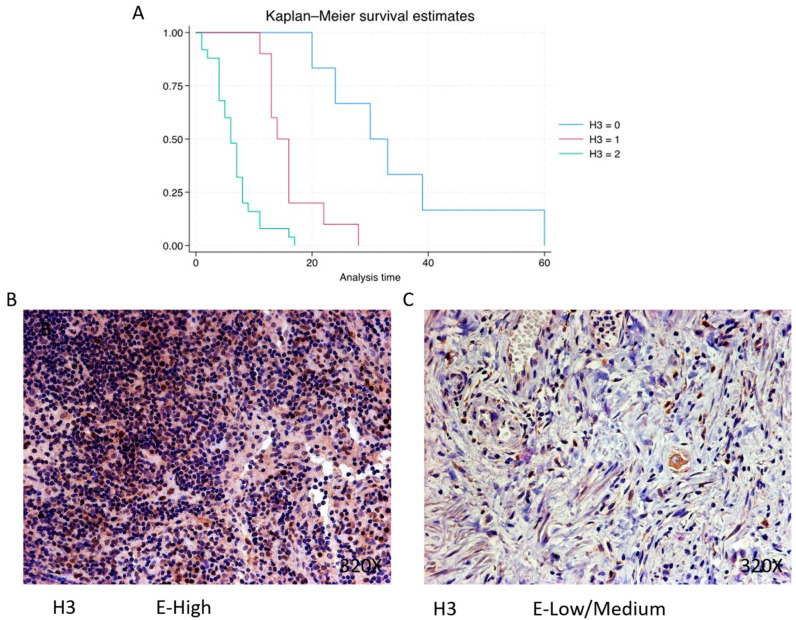
(**A**) Kaplan–Meier curves for survival time according to protein expression of H3. Blue curve = negative expression; red curve = low/medium expression; green curve = high expression. (**B**,**C**) Images showing histopathological expression of H3 in patients diagnosed with pancreatic cancer. High expression of the protein corresponds to an IRS score of 3, whereas low/medium expression corresponds to an IRS score of 1/2.

Histone 3 (H3) is predominantly expressed at a high level in 60.98% of cases, correlating with a median survival of 6 (4–8) months. The hazard ratio of 5.386, along with a 95% confidence interval [2.734, 10.609], indicates a substantial impact on survival (*p*-value < 0.001 ***). Negative expression is observed in 14.63% of cases with a median survival of 31.5 months, while low/moderate expression is seen in 24.39% with a median survival of 15 months. Kaplan–Meier survival estimates of H3 and examples of high versus low/medium expression are summarized in Figure 6A–C.

**Figure 7:**
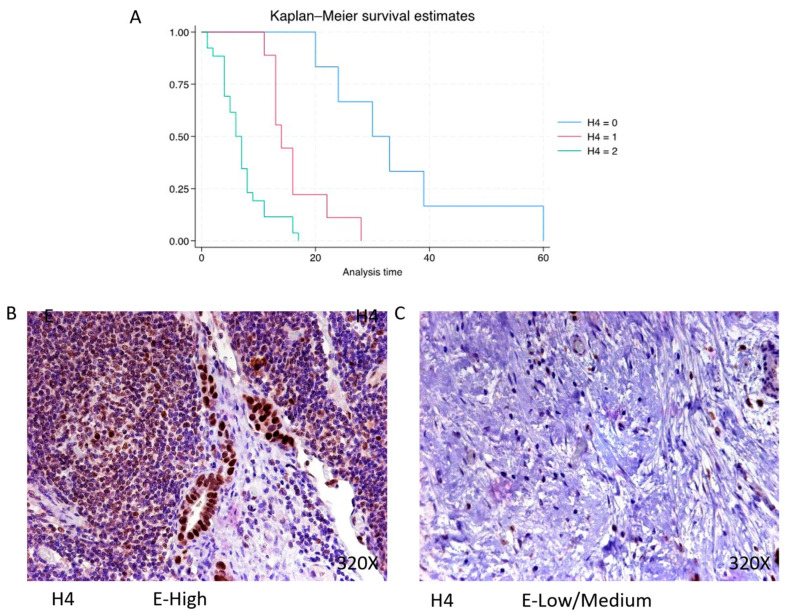
(**A**) Kaplan–Meier curves for survival time according to protein expression of H4. Blue curve = negative expression; red curve = low/medium expression; green curve = high expression. (**B**,**C**) Images showing histopathological expression of H4 in patients diagnosed with pancreatic cancer. High expression of the protein corresponds to an IRS score of 3, whereas low/medium expression corresponds to an IRS score of 1/2.

Histone 4 (H4) shows predominantly high expression in 63.42% of PDAC cases, with a median survival of 6.5 (4–8) months. The hazard ratio of 4.932, along with a 95% confidence interval [2.481, 9.802], signifies a significant impact on survival (*p*-value < 0.001 ***). Negative expression is observed in 14.63% of cases with a median survival of 31.5 months, while low/moderate expression is seen in 21.95% with a median survival of 14 months. Kaplan–Meier survival estimates of H4 and examples of high versus low/medium expression are summarized in Figure 7A–C.

**Figure 8:**
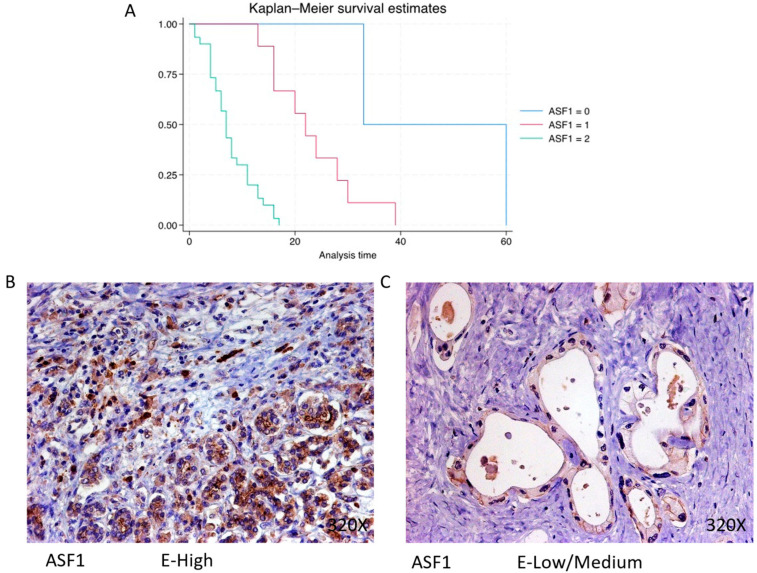
(**A**) Kaplan–Meier curves for survival time according to protein expression of ASF-1. Blue curve = negative expression; red curve = low/medium expression; green curve = high expression. (**B**,**C**) Images showing histopathological expression of ASF-1 in patients diagnosed with pancreatic cancer. High expression of the protein corresponds to an IRS score of 3, whereas low/medium expression corresponds to an IRS score of 1/2.

In PDAC, ASF-1 is predominantly expressed at a high level in 73.17% of cases, correlating with a median survival of 7 (4.25–11) months. The hazard ratio of 9.004, with a 95% confidence interval [3.161, 25.646], indicates a substantial impact on survival (*p*-value < 0.001 ***). Negative expression is observed in 4.88% of cases with a median survival of 46.5 months, while low/moderate expression is seen in 21.95% with a median survival of 22 months. Kaplan–Meier survival estimates of ASF-1 and examples of high versus low/medium expression are summarized in Figure 8A–C.

### 3.3. Increased Expression of the Metabolic Enzyme PGAM1 Is Associated with Decreased Survival in Pancreatic Cancer Patients

**Figure 9:**
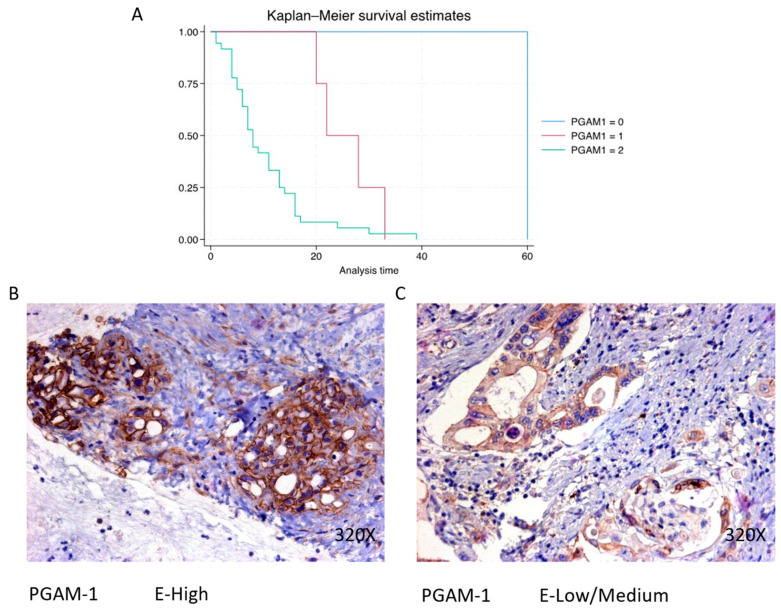
(**A**) Kaplan–Meier curves for survival time according to protein expression of PGAM1. Blue curve = negative expression; red curve = low/medium expression; green curve = high expression. (**B**,**C**) Images showing histopathological expression of PGAM1 in patients diagnosed with pancreatic cancer. High expression of the protein corresponds to an IRS score of 3, whereas low/medium expression corresponds to an IRS score of 1/2.

PGAM1 expression is predominantly high in 87.8% of PDAC cases, associated with a median survival of 8 (5–13.2) months. The hazard ratio of 3.681, with a 95% confidence interval [1.403, 9.661], signifies a significant impact on survival (*p*-value < 0.001 ***). Negative expression is observed in 2.44% of cases with a median survival of 60 months, while low/moderate expression is seen in 9.76% with a median survival of 25 months. Kaplan–Meier survival estimates of PGAM1 and examples of high versus low/medium expression are summarized in Figure 9A–C.

### 3.4. AIF-1, IL-12A and IL-18 Are Directly Related to Pancreatic Cancer Mortality, Whereas IL-10 Shows an Inverse Association

Finally, our analysis of inflammatory biomarkers shows that AIF-1, IL-12A and IL-18 are directly related to pancreatic cancer mortality, whereas IL-10 shows an inverse association.

**Figure 10:**
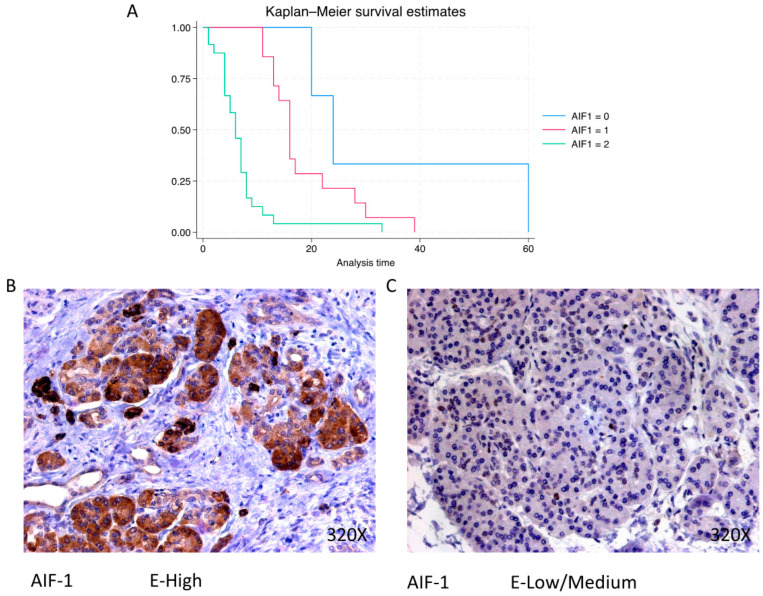
(**A**) Kaplan–Meier curves for survival time according to protein expression of AIF-1. Blue curve = negative expression; red curve = low/medium expression; green curve = high expression. (**B**,**C**) Images showing histopathological expression of AIF-1 in patients diagnosed with pancreatic cancer. High expression of the protein corresponds to an IRS score of 3, whereas low/medium expression corresponds to an IRS score of 1/2.

AIF-1 shows a predominantly high expression in 58.54% of cases, correlating with a median survival of 6 (4–8) months. The hazard ratio of 3.703, with a 95% confidence interval [2.005, 6.837], indicates a substantial impact on survival (*p*-value < 0.001 ***). Negative expression is observed in 7.32% of cases with a median survival of 24 months, while low/moderate expression is seen in 34.15% with a median survival of 16 months. Kaplan–Meier survival estimates of IPO-5 and examples of high versus low/medium expression are summarized in Figure 10A–C.

**Figure 11:**
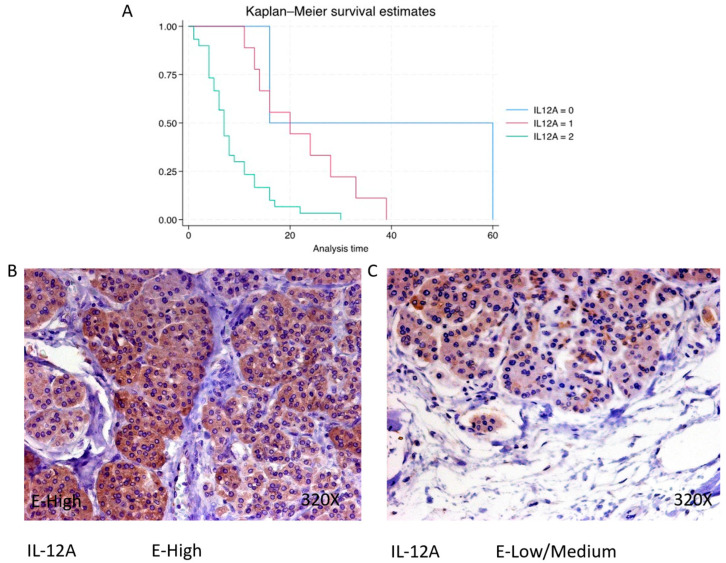
(**A**) Kaplan–Meier curves for survival time according to protein expression of IL-12A. Blue curve = negative expression; red curve = low/medium expression; green curve = high expression. (**B**,**C**) Images showing histopathological expression of IL-12A in patients diagnosed with pancreatic cancer. High expression of the protein corresponds to an IRS score of 3, whereas low/medium expression corresponds to an IRS score of 1/2.

IL-12A expression is predominantly high in 73.17% of PDAC cases, with a median survival of 7 (4.25–11) months. The hazard ratio of 3.473, with a 95% confidence interval [1.695, 7.114], signifies a significant impact on survival (*p*-value = 0.001 **). Negative expression is observed in 4.88% of cases with a median survival of 38 months, while low/moderate expression is seen in 21.95% with a median survival of 20 months. Kaplan–Meier survival estimates of IL-12A and examples of high versus low/medium expression are summarized in Figure 11A–C.

**Figure 12:**
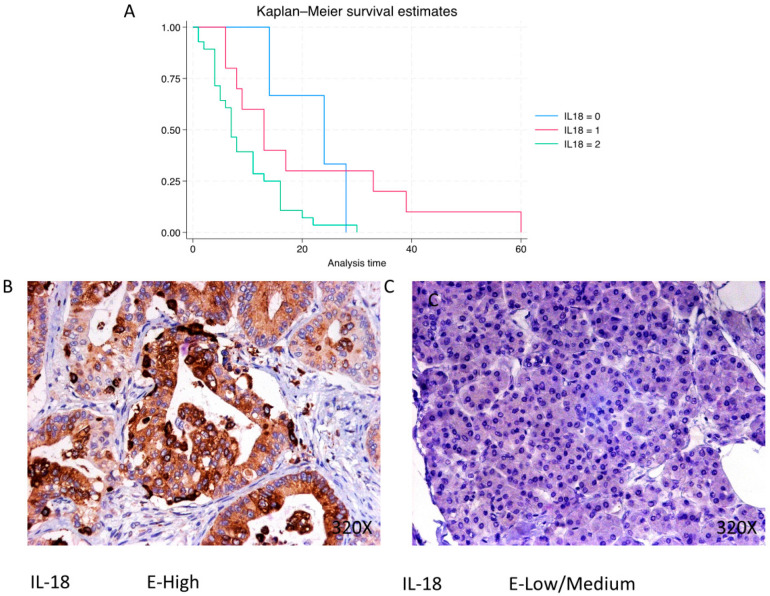
(**A**) Kaplan–Meier curves for survival time according to protein expression of IL-18. Blue curve = negative expression; red curve = low/medium expression; green curve = high expression. (**B**,**C**) Images showing histopathological expression of IL-18 in patients diagnosed with pancreatic cancer. High expression of the protein corresponds to an IRS score of 3, whereas low/medium expression corresponds to an IRS score of 1/2.

In PDAC, IL-18 is predominantly expressed at a high level in 68.29% of cases, with a median survival of 7 (4–13.8) months. The hazard ratio of 1.958, with a 95% confidence interval [1.100, 3.485], indicates a significant impact on survival (*p*-value = 0.022 *). Negative expression is observed in 7.32% of cases with a median survival of 24 months, while low/moderate expression is seen in 24.39% with a median survival of 13 months. Kaplan–Meier survival estimates of IL-18 and examples of high versus low/medium expression are summarized in Figure 12A–C.

**Figure 13:**
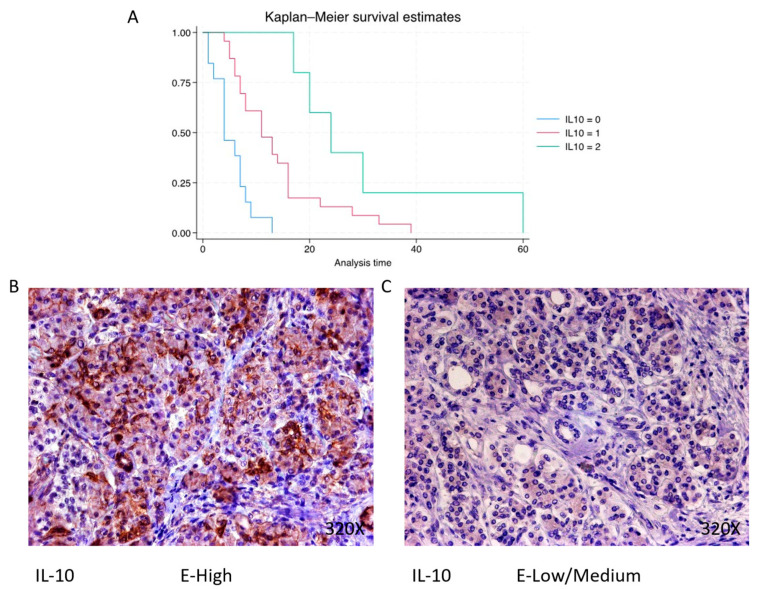
(**A**) Kaplan–Meier curves for survival time according to protein expression of IL-10. Blue curve = negative expression; red curve = low/medium expression; green curve = high expression. (**B**,**C**) Images showing histopathological expression of IL-10 in patients diagnosed with pancreatic cancer. High expression of the protein corresponds to an IRS score of 3, whereas low/medium expression corresponds to an IRS score of 1/2.

Finally, immunohistochemical analysis shows that IL-10 is expressed in 68.3% of pancreatic cancer patients, whereas 31.7% present negative expression for this cytokine. The median survival for patients with pancreatic cancer and negative tissue expression of IL-10 was 4 (4–7) months. However, in the case of patients with low–medium expression, it was 11 (7–16) months, increasing to 24 (20–30) months in patients with a high expression (Figure 2B). Global comparisons showed that the significance value was *p* < 0.001 ***, with a hazard ratio of 0.265 and a 95% confidence interval [0.142–0.500] in patients with high expressions of IL-10. Kaplan–Meier survival estimates of IL-10 and examples of high versus low/medium expression are summarized in Figure 13A–C.

### 3.5. Correlation Analysis

**Figure 14:**
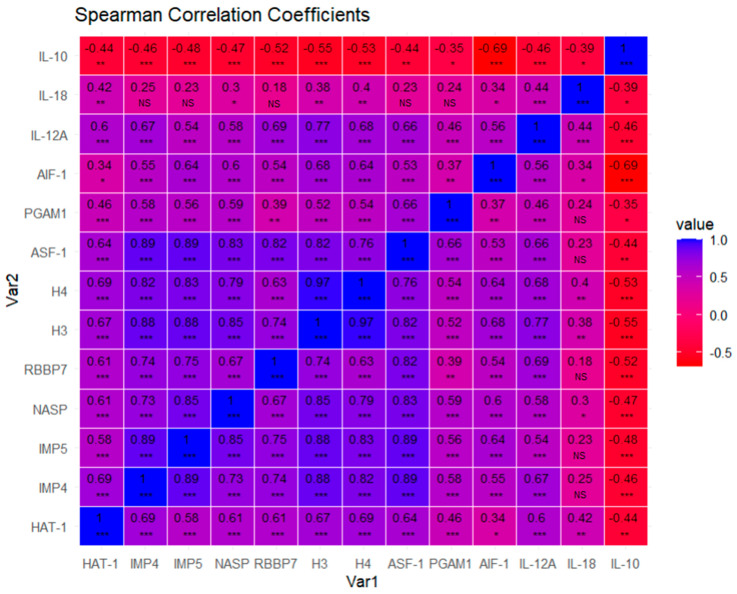
Pairwise scatterplot matrix with Spearman correlation coefficients. *p*-values associated with each Spearman correlation coefficient are noted with asterisks and were adjusted via false discovery rate correction (FDR): adjusted alpha (0.001; 0.01; 0.05): 0.00400000 ***; 0.02000000 **; 0.06666667 *. NS = Non significative. The exact *p*-value associated with each Spearman correlation coefficient for each pair of variables can be found in the Supplementary Materials (SM1).

Through a correlation analysis, we could observe how proteins are correlated with the help of matrix correlation expressed by Spearman coefficients and a heatmap (Figure 14). We visualized how most of the biomarkers are positively correlated, except anti-inflammatory cytokine IL-10, which is negatively correlated, with all of them being statistically significant for the vast majority of cases, except for IL-18, which correlates with less proteins and with less strength or significance. We observe a pattern of highly correlated proteins, suggesting functional relationships. Therefore, we may conclude that these molecular markers tend to be co-expressed, providing insights into how these proteins interact or influence each other’s expression levels.

## 4. Discussion

In the present study, a Kaplan–Meier analysis was conducted to correlate the expression levels of epigenetic modulation markers (H3/H4, HAT-1, ASF1, NASP, RBBP7, IPO4, IPO5), metabolic regulators (PGAM), and inflammatory mediators (AIF-1, IL-10, IL-12A, and IL-18) with the survival of patients diagnosed with PDAC. Generally, an inversely proportional trend is observed between the expression of these components and the survival rate, indicating a higher mortality is associated with elevated expression levels, with the exception of IL-10, for which an opposite relationship is observed. Additionally, a correlographic analysis was performed, revealing direct associations among the expressions of the various components mentioned, with the sole exception of IL-10, which exhibits an inverse relationship with the other elements studied. These findings raise important questions about the underlying mechanisms and the possible functional implication of all these molecules in the progression and prognosis of PDAC, requiring a deeper and specific analysis to understand their role in this clinical context.

Regarding epigenetic modulators, we selected the following biomarkers to be explored: H3/4, HAT-1, ASF1, NASP, RBBP7, IPO4, IP5. All these markers exhibit a direct relationship between their expression level and a reduced survival in PADC patients. However, HAT-1 seemed to present the clearest relationship between its expression and cancer mortality, with a hazard risk of 21.74. The relevance of HAT-1 in cancer has been consistently demonstrated in the available literature [25]. In pancreatic tumors, HAT1 has shown its ability for influencing tumor proliferation and immunity, as well as treatment resistance, supporting its potential role as a relevant diagnostic, prognostic and predictive marker for cancer therapy [26,27]. HAT-1 is a molecule mainly implicated in the enzymatic acetylation of K5 and K12 in the N-terminus of H4 in a newly assembled H3/H4 dimer in the cytoplasm [28]. However, HAT-1 also fulfills a wide range of non-canonical actions, including translocation of H3H4 into the nucleus; regulation of H4 gene expression; DNA replication fork stability and replication-coupled chromatin assembly; DNA damage repair and telomeric silencing; epigenetic regulation of nuclear lamina-associated heterochromatin; and promoting acetylation and succinylation processes [29]. HAT-1 first interacts and forms a complex with RBBP7, leading to the acetylation of the H3/H4 heterodimer as previously mentioned. Then, the acetylated H3/H4 dimers interact with the chaperone ASF-1 participating in the transport of the HAT1/RBBP7/H3/H4 complex from the cytoplasm to the nucleus via importins like IPO4 and IPO5 [30,31,32]. Subsequently, this complex interacts with the chromatin assembly factor 1 and the proliferating cell nuclear antigen, thus facilitating the pathway for deposition of newly synthesized histones to regulate gene transcription [25]. In a different chromatin assembly process, as a replacement of Asf1, HAT1 can bind to NASP, another histone chaperone [32]. Overall, the complex formed by HAT1, H3/H4, RBBP7 and ASF1/NASP modulates critical target genes to influence in cell metabolism, DNA repair, cell cycle and death and molecular signaling [25]. In our study, we have also observed that higher expression levels of these proteins related to HAT-1 (H3/H4, ASF-1, NASP and RBBP7) are directly correlated to a reduced survival, suggesting that this molecular network might represent an important part related to PDAC progression. The relevance of these proteins as prognostic factors for oncological patients has been demonstrated previously [33,34,35,36]. In the event of PDAC, these molecules have been shown to be implicated in a broad molecular network that facilitates pancreatic cancer progression [33,37], which may explain the association of these variables with a poor prognosis.

On the other hand, PGAM-1 is a crucial glycolytic enzyme that catalyzes the conversion of 3-phosphoglycerate to 2-phosphoglycerate. Overexpression of PGAM has been reported in several types of tumors, enhancing the glycolytic flux of tumoral cells in detriment of oxidative phosphorylation, leading to a metabolic reprogramming phenomenon known as the Warburg effect [38]. The relevance of aerobic glycolysis or the Warburg effect in the progression of pancreatic cancer is consistently supported by the available evidence [39]. Indeed, past works have shown a direct relationship between the protein expression of PGAM1 and reduced survival in PDAC patients [40,41]. HAT1 seems to present an important succinyl transferase activity in histones and non-histone proteins like PGAM1 [29]. The succinylation of PGAM1 by HAT1 seems to play a critical role in promoting tumor progression in vitro and in vivo in various types of cancer, including PDAC [42].

Additionally, our results show a direct correlation between various inflammatory markers (AIF-1, IL-12A and IL-18) and reduced survival, whereas IL-10 exhibited an inverse correlation. AIF-1 is a cytosolic protein with a molecular weight of 17 kDa, encoded in the HLA class III genomic region, which exhibits calcium- and actin-binding properties, with its expression being elicited by cytokines such as interferon gamma (IFN-γ) [43]. This molecule orchestrates both immune (i.e., controlling proliferation, the migration and polarization of T lymphocytes, and the activation of macrophages or microglia or modulating dendritic cells) and non-immune actions (i.e., modulation of membrane ruffling, activation of endothelial cells, cell cycle progression and F-actin binding activity) [44]. The role of AIF-1 in cancer has been demonstrated in past works, mainly focused on hepatocellular carcinoma [45], breast [46], lung [47] and other types of cancer [48,49,50]. To our best knowledge, no studies have been conducted to understand the carcinogenic role and prognostic value of AIF-1 in pancreatic cancer; a recent study [51] revealed that AIF-1 acts as a robust diagnostic and prognostic biomarker in several types of cancer and is closely correlated with tumor immune infiltration. Therefore, our results suggest that AIF-1 could be an important prognostic marker and carcinogenic mechanism in PDAC, encouraging further studies in this field.

AIF-1 is activated under inflammatory conditions (mainly by IFN-γ as already mentioned) and in turn is responsible for the regulation of a wide variety of inflammatory mediators, including IL-10, IL-12A and IL-18 [52,53,54]. IL-12A and IL-18 are two major proinflammatory cytokines widely explored in different disease conditions [55,56,57]. IL-12 is a heterodimeric cytokine formed by IL-12A (also known as p35) and IL-12B (p40). This cytokine is mainly produced by B cells, dendritic cells and macrophages and regulates IFN-γ production, T cell differentiation and other functions [58]. IL-18 also exerts a broad spectrum of immunomodulatory actions, but perhaps one of the most studied effects is related to the activation of inflammasomes like NLRP3 [59]. The pivotal role of IL-12 and IL-18 in cancer is also supported by previous studies [60,61]. In PDAC, the relevance of the IL-18 and NLRP3 inflammasomes has been supported by compelling evidence, suggesting that both components are interesting prognostic biomarkers [20,62,63]. Most studies have correlated serum levels IL-18 with overall survival in PDAC patients, with heterogenic results [63]. A lower number of studies focusing on tissue expression of IL-18 have also shown that higher IL18 in pancreatic cancer tissues is associated with shorter OSs and increased invasion and metastasis [64]. Despite fewer studies, previous works have also found that pancreatic carcinoma cells not only produce IL-18, but also IL-12, and that higher serum and tissue levels of IL-12 can be found in pancreatic cancer cells [65]. On the other hand, IL-10 is one of the best-characterized anti-inflammatory cytokines, with a pivotal role in the limitation of inflammatory responses and in the maintenance of tissue homeostasis [66]. The role of IL-10 in cancer is still controversial. IL-10 is produced by a wide variety of cells and it exhibits highly pleiotropic effects that may play a dual role in cancer initiation and development [67]. In the case of PDAC, most studies have found that PDAC patients commonly present increased levels of circulating IL-10 [68]. In addition, it seems that higher circulating levels of IL-10 appear to be associated with a reduced survival [69,70]. In this study, we have observed that higher IL-10 tissue levels are associated with increased survival. Despite the fact that the prognostic role of IL-10 in histopathological samples is less established, Bellone et al. [65] reported that patients with PDAC tend to present higher levels of IL-10, although they did not find any association between its expression and survival. Zhao et al. [71] demonstrated that IL10 could inhibit the growth of a transplanted pancreatic tumor in vivo and prolong the survival of mice, mainly through the indirect inhibition of the secretion of pro-inflammatory cytokines IL6 and TNF-α and tumor angiogenesis formation. The immunomodulatory role that IL-10 exerts in PDAC could partially explain our results, although further studies are warranted before drawing significant conclusions.

Finally, the tight correlations observed for almost all proteins examined in the correlation plot highlight the outstanding interactions between these molecular entities. The results reveal a possible coordination of gene regulation, suggesting that these proteins are involved in common pathways or cellular processes. Such intricate interaction networks may contribute to the coordination of complex biological functions, underlining the need to further study the molecular and functional relationships of these proteins. It is also true that the greatest level of correlations is observed between well-established biological networks such as HAT1/IPO4/IPO5/H3/H4/NASP/RBBP7/ASF1/PGAM1 or AIF-1/IL-10/IL-12A. IL-18 is the component with the lowest level of correlation with the other components. The observed associations may have clinical implications that can influence diagnostic and prognostic strategies or therapeutic interventions in different forms that could be explored in future works. However, it is important to acknowledge the limitations of this study, including the need for validation in other cohorts and the need to consider potential confounding factors. Addressing these limitations and pursuing future research directions, such as investigating specific biological contexts and elucidating the functional consequences of these correlations, will help improve our understanding of the complex molecular landscape revealed by this comprehensive correlation map analysis.

## 5. Conclusions

In this work, we have demonstrated the prognostic value of a set of epigenetic (H3/H4, HAT-1, ASF1, NASP, RBBP7, IPO4, IPO5), metabolic (PGAM), and inflammatory biomarkers (AIF-1, IL-10, IL-12A, and IL-18) in 41 patients diagnosed with and who passed away due to PDAC. Higher expression levels of these molecules were directly associated with an increased PDAC mortality, except in the case of IL-10, for which an inverse association was reported. HAT-1 was identified as the molecule most distinctly associated with mortality, further emphasizing the importance of specific markers in prognostic assessments.

**Figure 15:**
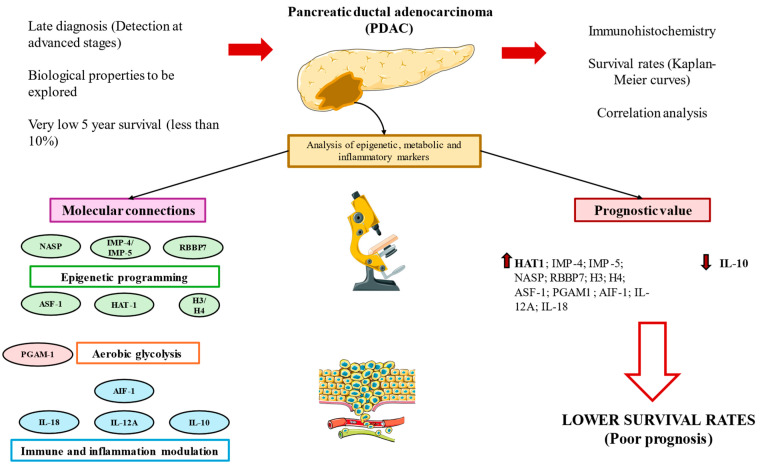
A graphical summary of the study. Pancreatic ductal adenocarcinoma (PDAC) is associated with a very poor prognosis due to its late diagnosis and the unknown biological characteristics of these tumors. In this study, we conducted immunohistochemistry and correlation analyses and constructed Kaplan–Meier curves to further explore the role and connections of various molecules implicated in epigenetic programming, aerobic glycolysis and immunoinflammatory modulation, which are indeed three hallmarks of cancer. Our results show that these molecules are implicated in the carcinogenesis of PDAC, and their expression levels are directly correlated, except for IL-10, which shows an inverse association. Increased expressions (up arrows) of almost all of these molecules are associated with a poor prognosis in PDAC, remarking the notable prognostic value of HAT-1. IL-10 was the only marker inversely associated with mortality (down arrows)

The correlogram analysis shed light on the complex network that underpins the development of pancreatic cancer and offered insightful information about possible connections among the chemicals under investigation. Figure 15 summarizes the main results obtained in this work. Although our findings greatly advance our knowledge of the PDAC prognostic environment, more research is clearly needed. Extensive research is necessary to validate the predictive significance of these elements, clarify the molecular pathways linking them and investigate the viability of focused therapy approaches. Understanding the molecular nuances of pancreatic cancer will help us to develop more robust diagnostic and treatment approaches that could enhance patient outcomes and lessen the severity of this dangerous illness.

## Figures and Tables

**Table 1:** Primary and secondary antibodies used and their dilutions.

Antigen	Species	Dilution	Provider	Protocol Specification
HAT-1	Rabbit monoclonal	1:1000	Abcam Cambridge, UK (ab193097)	100% Triton 0.1% in PBS, 10 min, before incubation with blocking solution
IPO-4	Rabbit monoclonal	1:100	Abcam (ab181046)	10 mM sodium citrate pH = 6 before incubation with blocking solution
IPO-5	Rabbit polyclonal	1:100	Abcam (ab187175)	100% Triton 0.1% in PBS, 10 min, before incubation with blocking solution
NASP	Mouse monoclonal	1:100	Santa Cruz Biotechnology, Santa Cruz, CA, USA (sc.514669)	10 mM sodium citrate pH = 6 before incubation with blocking solution
RBBP7	Rabbit monoclonal	1:2000	Abcam (ab259957)	10 mM sodium citrate pH = 6 before incubation with blocking solution
H3	Rabbit polyclonal	1:100	Abcam (ab1791)	100% Triton 0.1% in PBS, 10 min, before incubation with blocking solution
H4	Rabbit monoclonal	1:500	Abcam (ab51997)	EDTA pH = 9 before incubation with blocking solution
ASF-1	Rabbit polyclonal	1:100	Abcam (ab235358)	100% Triton 0.1% in PBS, 10 min, before incubation with blocking solution
PGAM-1	Rabbit monoclonal	1:25	Abcam (ab247037)	EDTA pH = 9 before incubation with blocking solution
AIF-1	Goat polyclonal	1:500	Abcam (ab5076)	EDTA pH = 9 before incubation with blocking solution
IL-12A	Rabbit monoclonal	1:100	Abcam (ab131039)	EDTA pH = 9 before incubation with blocking solution
IL-18	Rabbit monoclonal	1:250	Abcam (ab243091)	10 mM sodium citrate pH = 6 before incubation with blocking solution
IL-10	Rabbit Polyclonal	1:100	Abcam (ab217941)	100% Triton 0.1% in PBS, 10 min, before incubation with blocking solution

**Table 2:** Examination of the clinical and sociodemographic features of patients who had been diagnosed with pancreatic cancer; n = number of patients and IQR = interquartile range.

**Age (Median [IQR])**	72.00 [45.00–88.00]
**Sex (n (%))**	
**Men**	27 (65.85%)
**Women**	14 (34.15%)
**Smoking habits**	18 (43.90%)
**Drinking habits**	11 (26.83%)
**Obesity**	2 (4.88%)
**Type 2 diabetes mellitus**	15 (55.56%)
**Chronic maladies**	4 (9.76%)
**Previous malignancies**	11 (26.83%)

## Data Availability

The datasets used and/or analyzed during the present study are available from the corresponding author upon reasonable request.

## Supplementary Materials

The following supporting information can be downloaded at: https://www.mdpi.com/article/10.3390/cimb46050239/s1, SM1. List of *p*-values associated with each Spearman correlation coefficient for each pair of variables.

## Appendix A

**Table A1:** Percentage of patients with different tumor stages and plasma levels of the main carcinogenic markers, collected routinely and expressed as a median and interquartile range.

**Tumor stage 4**	31.71% (13/41)
**Tumor stage ≤ 4**	68.29% (28/41)
**Median levels of** **Ca 19.9 U/mL (0–37)**	102.10 [44.91–805.00]
**Median levels of** **CEA ng/mL (0–5)**	5.43 [2.71–11.31]
**Median levels of** **AFP ng/mL (0–13.4)**	2.32 [1.46–4.39]

**Table A2:** Median survival values and rate estimation for each biomarker.

Molecular Marker	Level of Expression	N = 41 n (%):	Median Survival	Q1–Q3	Hazard Ratio	Confidence Interval	*p* Value
HAT-1	Negative	1 (2.44)	60	60–60			
	Low/Moderate	6 (14.63)	29	25–32.2	21.743	[2.908621–162.5435]	0.003 **
	High	34 (82.93)	7.5	5–13			
IMP-4	Negative	1 (2.44)	60	60–60			
	Low/Moderate	12 (29.27)	21	15.2–28.5	8.094	[3.080211–21.26741]	<0.001 ***
	High	28 (68.29)	7	4–9.5			
IMP-5	Negative	2 (4.88)	45	37.5–52.5			
	Low/Moderate	11 (26.83)	20	16–26	7.493	[3.066998–18.30788]	<0.001 ***
	High	28 (68.29)	7	4–9.5			
NASP	Negative	2 (4.88)	45	37.5–52.5			
	Low/Moderate	10 (24.39)	21	16–27	7.563	[2.927238–19.53798]	<0.001 ***
	High	29 (70.73)	7	4–11			
RBBP7	Negative	3 (7.32)	30	23–45			
	Low/Moderate	5 (12.2)	24	20–33	4.024	[1.787804–9.058966]	<0.001 ***
	High	33 (80.49)	7	5–13			
H3	Negative	6 (14.63)	31.5	25.5–37.5			
	Low/Moderate	10 (24.39)	15	13–16	5.386	[2.734019–10.60884]	<0.001 ***
	High	25 (60.98)	6	4–8			
H4	Negative	6 (14.63)	31.5	25.5–37.5			
	Low/Moderate	9 (21.95)	14	13–16	4.932	[2.481096–9.801876]	<0.001 ***
	High	26 (63.42)	6.5	4–8			
ASF-1	Negative	2 (4.88)	46.5	39.8–53.2			
	Low/Moderate	9 (21.95)	22	16–28	9.004	[3.161273–25.64641]	<0.001 ***
	High	30 (73.17)	7	4.25–11			
PGAM1	Negative	1 (2.44)	60	60–60			
	Low/Moderate	4 (9.76)	25	21.5–29.2	3.681	[1.402629–9.661442]	<0.001 ***
	High	36 (87.8)	8	5–13.2			
AIF-1	Negative	3 (7.32)	24	22–42			
	Low/Moderate	14 (34.15)	16	13.2–20.8	3.703	[2.005442–6.836674]	<0.001 ***
	High	24 (58.54)	6	4–8			
IL-12A	Negative	2 (4.88)	38	27–49			
	Low/Moderate	9 (21.95)	20	14–28	3.473	[1.694996–7.11442]	0.001 **
	High	30 (73.17)	7	4.25–11			
IL-18	Negative	3 (7.32)	24	19–26			
	Low/Moderate	10 (24.39)	13	8.25–29	1.958	[1.100421–3.485366]	0.022 *
	High	28 (68.29)	7	4–13.8			
IL-10	Negative	13 (31.7)	4	4–7			
	Low/Moderate	23 (56.1)	11	7–16	0.265	[0.1416624–0.4964779]	<0.001 ***
	High	5 (12.2)	24	20–30			

Median and interquartile range (Q1–Q3) of each molecular parameter for the different groups of patients (level of expression). Hazard ratio given with *p* value significance: <0.05 *, <0.01 **, <0.001 ***.
