# Supplementary material for: Prognostic Value of Histone Acetyl Transferase 1 (HAT-1) and Inflammatory Signatures in Pancreatic Cancer
Source: Curr Issues Mol Biol. 2024 Apr 25;46(5):3839–65. doi: 10.3390/cimb46050239 (PMC11119917; doi:10.3390/cimb46050239)
Supplement: Supplementary file 1 [file cimb-46-00239-s001.zip › cimb-2962552-supplementary.pdf]

Supplementary material (SM):

SM1. List of p-values associated to each Spearman correlation coefficient for each pair of variables:

```
$`HAT-1-IMP4`
[1] 6.787995e
-07

$`HAT-1-IMP5`
[1] 8.111142e
-05

$`HAT-1-NASP`
[1] 2.621481e
-05

$`HAT-1-RBBP7
`
[1] 2.784122e
-05

$`HAT-1-H3`
[1] 1.503178e
-06

$`HAT-1-H4`
[1] 6.227679e
-07

$`HAT-1-ASF-1
`
[1] 6.378745e
-06

$`HAT-1-PGAM1
`
[1] 0.0027579
94

$`HAT-1-AIF-1
`
[1] 0.0288277
5

$`HAT-1-IL-12
A`
[1] 3.024147e
-05

$`HAT-1-IL-18
`
[1] 0.0059357
72

$`HAT-1-IL-10
`
[1] 0.0040999
83

$`IMP4-IMP5`
[1] 5.329071e
-15

$`IMP4-NASP`
[1] 5.382798e
-08

$`IMP4-RBBP7`
[1] 4.198317e
-08

$`IMP4-H3`
[1] 6.350476e
-14

$`IMP4-H4`
[1] 5.312062e
-11

$`IMP4-ASF-1`
[1] 5.329071e
-15

$`IMP4-PGAM1`
[1] 7.221345e
-05

$`IMP4-AIF-1`
[1] 0.0002055
784

$`IMP4-IL-12A
`
[1] 1.318041e
-06

$`IMP4-IL-18`
[1] 0.1224245

$`IMP4-IL-10`
[1] 0.0022692
44

$`IMP5-NASP`
[1] 2.615241e
-12

$`IMP5-RBBP7`
[1] 1.28738e-
08

$`IMP5-H3`
[1] 3.952394e
-14

$`IMP5-H4`
[1] 3.135536e
-11

$`IMP5-ASF-1`
[1] 5.329071e
-15

$`IMP5-PGAM1`
[1] 0.0001525
647

$`IMP5-AIF-1`
[1] 6.358696e
-06

$`IMP5-IL-12A
`
[1] 0.0002626
645

$`IMP5-IL-18`
[1] 0.1527061

$`IMP5-IL-10`
[1] 0.0015363
68

$`NASP-RBBP7`
[1] 1.442853e
-06

$`NASP-H3`
[1] 2.282619e
-12

$`NASP-H4`
[1] 7.709597e
-10

$`NASP-ASF-1`
[1] 1.808265e
-11

$`NASP-PGAM1`
[1] 5.621866e
-05

$`NASP-AIF-1`
[1] 3.248003e
-05

$`NASP-IL-12A
`
[1] 6.560214e
-05

$`NASP-IL-18`
[1] 0.0566136
4

$`NASP-IL-10`
[1] 0.0020986
42

$`RBBP7-H3`
[1] 4.347481e
-08

$`RBBP7-H4`
[1] 8.222441e
-06

$`RBBP7-ASF-1
`
[1] 3.398881e
-11

$`RBBP7-PGAM1
`
[1] 0.0127835
8

$`RBBP7-AIF-1
`
[1] 0.0002355
17

$`RBBP7-IL-12
A`
[1] 7.616569e
-07

$`RBBP7-IL-18
`
[1] 0.2643393

$`RBBP7-IL-10
`
[1] 0.0004923
455

$`H3-H4`
[1] 0

$`H3-ASF-1`
[1] 5.208722e
-11

$`H3-PGAM1`
[1] 0.0004912
666

$`H3-AIF-1`
[1] 1.177401e
-06

$`H3-IL-12A`
[1] 4.419348e
-09

$`H3-IL-18`
[1] 0.0156517
7

$`H3-IL-10`
[1] 0.0002181
515

$`H4-ASF-1`
[1] 1.027372e
-08
```

```
$`H4-PGAM1`
[1] 0.0003051
626

$`H4-AIF-1`
[1] 7.521702e
-06

$`H4-IL-12A`
[1] 9.02059e-
07

$`H4-IL-18`
[1] 0.0087478
04

$`H4-IL-10`
[1] 0.0003611
978

$`ASF-1-PGAM1
`
[1] 2.403285e
-06

$`ASF-1-AIF-1
`
[1] 0.0003221
601

$`ASF-1-IL-12
A`
[1] 2.829045e
-06

$`ASF-1-IL-18
`
[1] 0.1403787

$`ASF-1-IL-10
`
[1] 0.0043851
62

$`PGAM1-AIF-1
`
[1] 0.0165192
3

$`PGAM1-IL-12
A`
[1] 0.0022544
15

$`PGAM1-IL-18
`
[1] 0.1230768

$`PGAM1-IL-10
`
[1] 0.0262647
9

$`AIF-1-IL-12
A`
[1] 0.0001353
335

$`AIF-1-IL-18
`
[1] 0.0319083
3

$`AIF-1-IL-10
`
[1] 5.19369e-
07

$`IL-12A-IL-1
8`
[1] 0.0035664
31

$`IL-12A-IL-1
0`
[1] 0.0027526
17

$`IL-18-IL-10
`
[1] 0.0124164
8
```
